# A High-Pressure Bi-Directional Cycloid Rotor Flowmeter

**DOI:** 10.3390/s140815480

**Published:** 2014-08-21

**Authors:** Shuo Liu, Fan Ding, Chuan Ding, Zaipeng Man

**Affiliations:** The State Key Lab of Fluid Power Transmission and Control, Zhejiang University, Hangzhou 310027, China; E-Mails: fding@zju.edu.cn (F.D.); dc1986@zju.edu.cn (C.D.); manzaipeng@126.com (Z.M.)

**Keywords:** flow measurement, positive displacement meter, cycloid rotor

## Abstract

The measurement of the flow rate of various liquids and gases is critical in industrial automation. Rotary positive displacement meters (rotary PD meters) are highly accurate flowmeters that are widely employed in engineering applications, especially in custody transfer operations and hydraulic control systems. This paper presents a high pressure rotary PD meter containing a pair of internal cycloid rotors. It has the advantages of concise structure, low pressure loss, high accuracy and low noise. The curve of the internal rotor is designed as an equidistant curtate epicycloid curve with the external rotor curve as its conjugate. The calculation method used to determine the displacement of the cycloid rotor flowmeter is discussed. A prototype was fabricated, and experiments were performed to confirm measurements over a flow range of 1–100 L/min with relative errors of less than ±0.5%. The pressure loss through the flowmeter was about 3 bar at a flow rate of 100 L/min.

## 1. Introduction

Flow rate is one of the most important parameters in industrial automation, and flowmeters are the most widely used instruments for measuring this parameter. Flowmeters can be divided into volumetric flowmeters and mass flowmeters. Although mass flowmeters, like Coriolis mass flowmeters, have developed rapidly in recent decades, volumetric flowmeters still play a large part in all kinds of flow measurement scenarios [1–3].

The rotary PD meter is considered to have a high accuracy and robustness [3]. Due to their many advantages, such as good stability, insensitivity to upstream velocity distribution and fluid viscosity, easy maintenance, rotary PD meters are still widely employed in engineering applications, especially in custody transfer operations and hydraulic control system [4,5]. Rotary PD meters require a fluid to mechanically displace components in the meter. Then the flow-rate can be measured by monitoring the rotation speed of the rotors [6].

Rotary PD meter suppliers have been using advanced manufacturing techniques and chronometry to produce more precise flowmeters [7]. Oval gear flowmeters offer small pressure losses and high accuracies, and accounted for the largest proportion of rotary PD meters around the world [3]. Much research has been done to improve the precision of oval gear flowmeters [8–10]. Vyskrebtsov pointed out that if a splitter is placed at the input to the measuring chamber, there will be eddy currents at the end clearances, which helps reduce internal leakage, but the disadvantages associated with imbalanced forces, including vibration, backlash, and excessive noise restrict the development of oval gear flowmeters.

**Figure 1.**
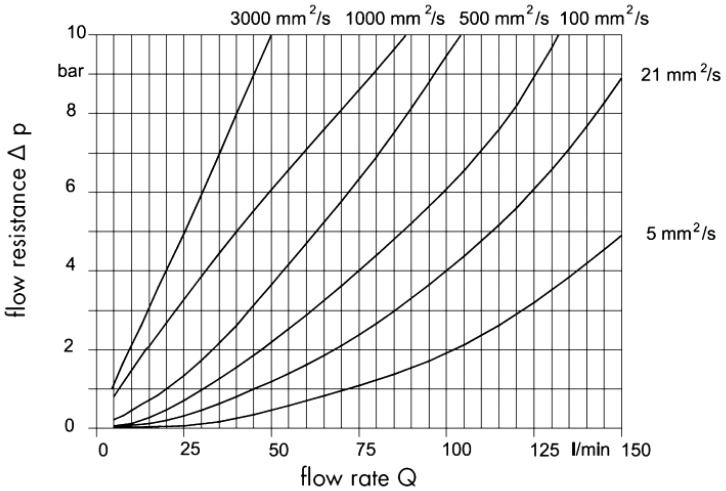
Pressure loss curves of a gear PD meter.

The rotary PD meter with a pair of profile shifted gears as its rotors is another kind of widely used flowmeter, which is very common in high pressure hydraulic control systems because of its wide measurement range and high accuracy [11,12]. However, the displacement of this kind of PD meter is so small that the rotor speed is too high and the pressure loss can be unbearable in some circumstances. Figure 1 presents the pressure loss curves of this kind of flowmeter [13]. When the kinematic viscosity of the oil is 100 cst and the flow rate is 100 L/min, the pressure loss is almost 6 bar. Another problem is that its high rotation speed under a large differential pressure shortens the bearing life.

It is a goal for designers to design a rotary PD meter with low pressure loss like the oval gear flowmeter and with small flow pulsation and noise like the profile shifted gear flowmeter. With the development of CAD and CAM, more complex helical screw rotors are being used in rotary PD meters. Because of the large displacement and helical screw rotors, this new type of PD meter has no flow pulsation and less pressure loss, but the excessive requirements for machining precision may lead to high cost and stuck rotors, which hinders its development [14,15].

Cycloid gears initially found applications as watch and clock gears. At present, such gears are applied in the Wankel engine, pumps and motors because they have many advantages such as large displacements, compact structures, and smooth running characteristics [16,17]. If the cycloid gear could be used in rotary PD meter, the performance of the rotary PD meter would be improved.

Since internal cycloid rotors can be designed with large eccentricity and few teeth, the displacement of the flowmeter with cycloid rotors is much larger than with profile shifted gear rotors, which will reduce the rotor speed and the pressure loss [3]. On the other hand, because the cycloid rotors have lower speed pulsation than oval gears, this type of flowmeter has lower noise and longer life than the oval gear flowmeters [18]. Furthermore, the profile curves of the cycloid rotors and the displacement can be accurately described by mathematical equations, which is helpful to simplify the flowmeter design process. Also, the easily machined curves of the rotors and the simplicity of the flowmeter structure reduce manufacturing costs dramatically.

This paper presents a high pressure bi-directional cycloid rotary PD meter, including the structure features, profile curves of the rotors and the displacement calculation method. A prototype was fabricated, and experiments were performed to examine the characteristics of the flowmeter.

## 2. Structure and Theoretical Analysis

### 2.1. Flowmeter Structure

**Figure 2.**
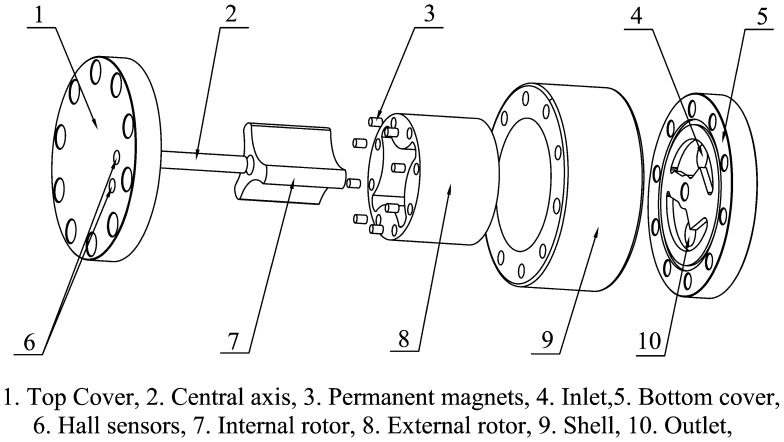
Structure of the high-pressure, bi-directional cycloid rotor flowmeter.

The structure of the proposed PD meter is quite simple, as shown in Figure 2. It is composed of a top cover, a bottom cover, a shell, internal and external rotors, a central axis, sensors, and bolts. The top cover, bottom cover, and shell comprise a sealed container that contains the external and internal rotors, which are the key components of the flowmeter. When the fluid enters the sealed container, the rotors rotate and divide the fluid into small packets. The flow rate is measured by counting the number of times the rotors rotate.

**Figure 3.**
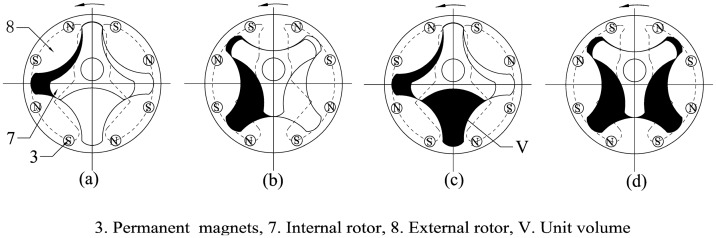
Rotation process of the flowmeter rotors.

The internal and external rotors are arranged eccentrically and joined to the stationary central axis and shell, respectively, with sliding bearings to guarantee that both rotors can rotate freely and simultaneously. The eccentricity should be as large as possible to ensure the maximization of the displacement. Several chambers are formed during the rotation of the rotors. At any time, the volumes of half of the chambers are expanding, whereas the volumes of the other half are decreasing. Two oil grooves need to be made in the bottom cover to maintain a continuous rotation. The inlet groove is used to connect all of the expanding-volume chambers and the inlet, whereas the outlet groove is used to connect all of the decreasing-volume chambers and the outlet. These connections are necessary to guarantee that no chamber can connect to the inlet and outlet grooves simultaneously regardless of where the rotors are. The oil groove shape used here is similar to the broken line shape shown in Figure 3. The same oil grooves are also machined in the top cover to eliminate unbalanced axial force on the rotors. As shown in Figure 3, the two rotors turn one third of a rotation from Figure 3a to 3c. During this time, one unit volume of fluid flows through the flowmeter.

The rotors in this cycloid rotor flowmeter are not axially restricted. The shell is designed to be a little taller than the rotors such that gaps are formed between the rotors and covers, allowing the rotors to float axially in the container. These gaps facilitate the formation of oil films to reduce friction between the rotors and covers, but the gap height must be extremely small (about 0.01 mm), because if the gap height increases a little, the internal leakage increases a lot. Because the gap is not between two surfaces but between two planes, its height can be controlled by using a surface grinder, whose machining precision can reach 0.01 mm.

**Figure 4.**
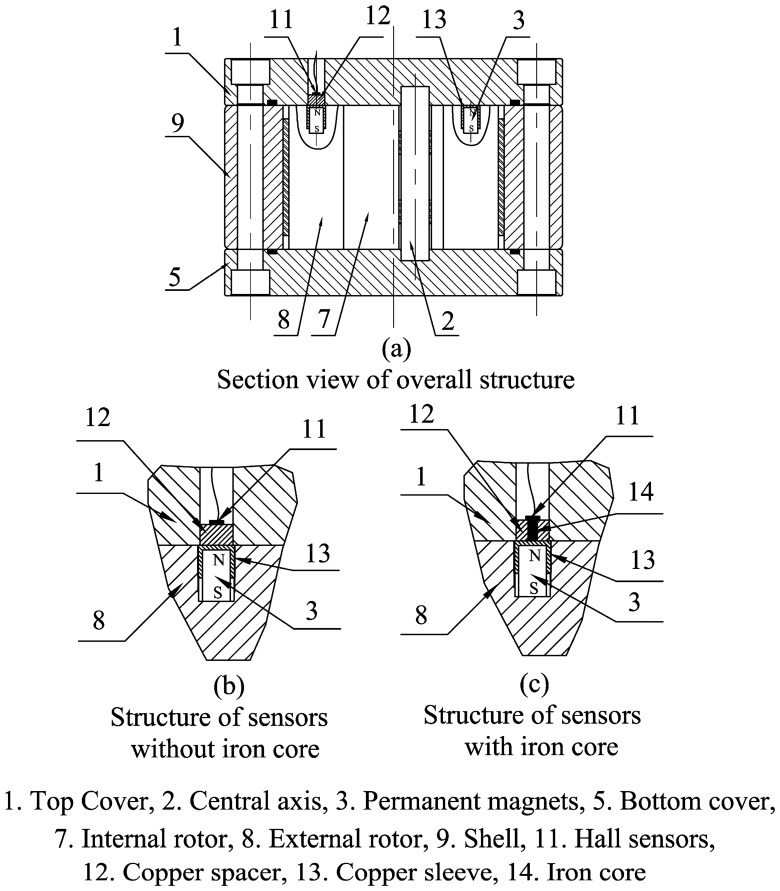
Structure of the sensors.

Figure 4 presents several sensors that have been designed to measure the rotational speed of the rotors. One cycloid rotor flowmeter has two Hall sensors built into the top cover and several pairs of permanent magnets in the external rotor. Magnetic isolation spacers are located under the Hall sensors and magnetic isolation sleeves outside the permanent permanents to enhance the magnetic field strength around the Hall sensors. Because the neighbouring permanent magnets have opposite polarities, an alternating magnetic field is generated in the Hall sensors when the rotors are in motion, as shown in Figure 3. Hence, after sending the signal through an appropriate shaping filter, one Hall sensor has a square wave output with the same frequency as the alternating magnetic field. In this research, four pairs of permanent magnets are uniformly distributed along the circumference of the external rotor, causing the Hall sensor to output four pulses for a single rotation, which makes the resolution is a quarter of the displacement.

The magnetic field strength around the Hall sensor can be further enhanced if a permeable iron core is set in the spacer, as shown in Figure 4c. The two sensors in the top cover are arranged at a certain angle so that they can output two square waves with different phases to facilitate the determination of the fluid direction from the phase change.

### 2.2. Description of Rotor Curves

The curve of the internal rotor is an equidistant curtate epicycloid curve, and the external rotor curve is its conjugate. In geometry, an epicycloid is a plane curve produced by tracing the path of a chosen point of a circle, called an epicycle, which rolls without slipping around a fixed circle. The point locus is regarded as a curtate epicycloid when the chosen point is inside the epicycle and as a prolate epicycloid when it is outside of the epicycle.

**Figure 5.**
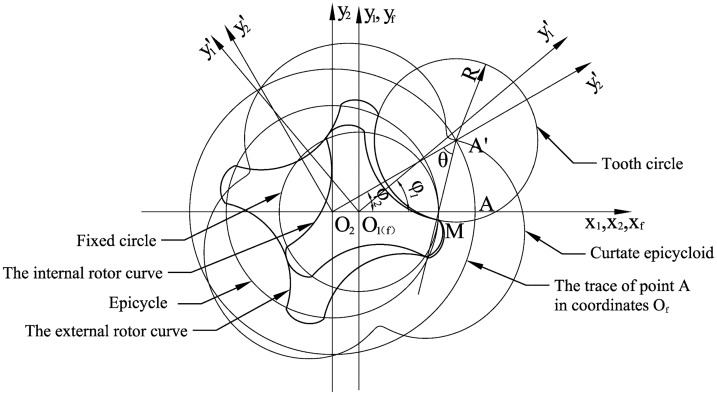
Established three-coordinate systems for the flowmeter rotors.

Three coordinate systems are established in Figure 5. Coordinate systems *O_1_* and *O_2_* are rotating coordinates with *O_1_* fixed to the base circle, *O_2_* fixed to a moving circle, and the two circles meeting each other at point *P*. Coordinate system *O_f_* is stationary. Point A is at the abscissa of *O_2_*; thus, the track of A in the coordinate system *O_1_* is the curtate epicycloid. The parametric equation of the curtate epicycloid is established using the complex vector method as follows:

(1) $$r_{\text{AO}1}(\varphi )=-a\cdot e^{-i\varphi_{1}}+L\cdot e^{i(\varphi_{2}-\varphi_{1})}$$

According to Figure 5, the radius of the tooth circle is *R*, and the centre of the tooth circle is at position *A*′ so that the equidistant curve must be meshed at point *M*. The trace of point *M* is the external rotor curve in the *O_2_* coordinate system and the internal rotor curve in the *O_1_* coordinate system.

The external rotor curve can be expressed by the following vector equation:

(2) $$r_{\text{MO}2}=L-R\cdot e^{i\theta }$$

where θ is a parameter that is changed over an interval such that the curve described by the equation is an arcing curve. The profile curve of an *n*-toothed external rotor contains *n* parts of arcing curves and *n* parts of transitional curves.

The equidistant curve is expressed as follows:

(3) $$r_{\text{MO}1}=L\cdot e^{i(\varphi_{2}-\varphi_{1})}-R\cdot e^{i(\theta +\varphi_{2}-\varphi_{1})}-a\cdot e^{-i\varphi_{1}}$$

Equation (3) can be transferred into Cartesian coordinates, which is the parametric equation of internal rotor:

(4) $$\left\{ \begin{array}{l} x=L\cos(\varphi_{2}-\varphi_{1})-R\cdot \cos(\theta +\varphi_{2}-\varphi_{1})-a\cdot \cos\varphi_{1}\\ y=L\sin(\varphi_{2}-\varphi_{1})-R\cdot \cos(\theta +\varphi_{2}-\varphi_{1})+a\cdot \sin\varphi_{1} \end{array}\right.$$

The profile curves of the rotors are described by mathematical equations easily, which is helpful to simplify the design process of the cycloid rotors flowmeter.

### 2.3. Displacement Calculation

In this section, the method for calculating the displacement of the flowmeter is described assuming that the flowmeter's internal rotors have three teeth and the external rotors have four teeth.

There has been empirical formula for calculating the displacement of cycloid rotor pump as follows:

(5) $$q_{\text{dis}}\approx \pi (r_{a1}^{2}-r_{f1}^{2})$$

But the results of this empirical formula have large errors—more than 5% when the internal rotor has three teeth. Obviously it is unacceptable for the design of a cycloid rotor flowmeter with a margin of error less than ±0.5%.

Considering one chamber between two adjacent mesh points, the kinetic equation of the internal rotor can be described using the conservation of energy equation as follows:

(6) $$P\frac{\text{dv}}{\text{dt}}=M_{i}\frac{d\varphi_{i}}{\text{dt}}+M_{o}\frac{d\varphi_{o}}{\text{dt}}$$

**Figure 6.**
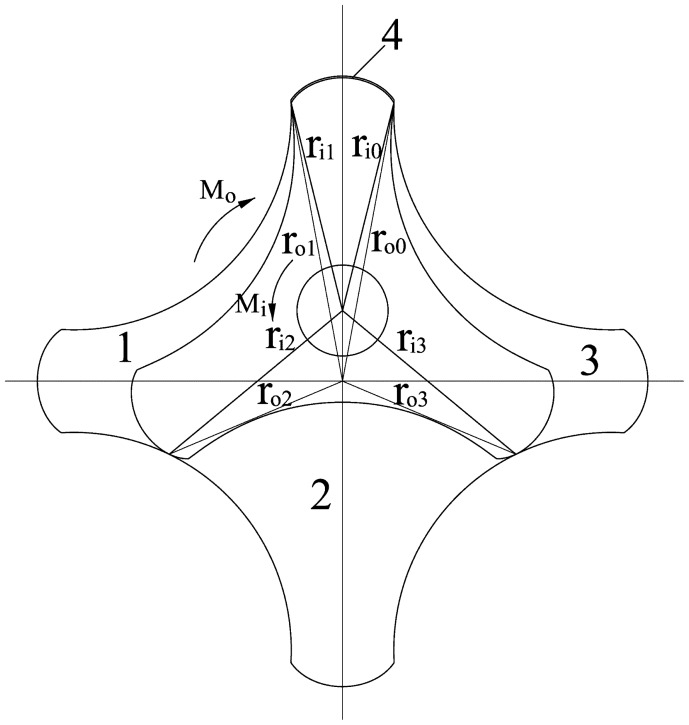
Torque applied to the external and internal rotors.

As shown in Figure 6, *M_i_* can be expressed as $M_{i}=\text{PB}\frac{\left({r_{i2}}^{2}-{r_{i1}}^{2}\right)}{2}$, and *M_o_* can be expressed as $M_{o}=\text{PB}\frac{\left({r_{o1}}^{2}-{r_{o2}}^{2}\right)}{2}$, where B is the thickness of the rotors. B is set to 1 to simplify the calculation.

The variable $\frac{\text{dv}}{\text{dt}}$ in Equation (6) is the instantaneous flow through one chamber and can be expressed as follows:

(7) $$\frac{\text{dv}}{\text{dt}}=\frac{\omega_{i}({r_{i2}}^{2}-{r_{i1}}^{2})}{2}+\frac{\omega_{o}({r_{o1}}^{2}-{r_{o2}}^{2})}{2}$$

**Figure 7.**
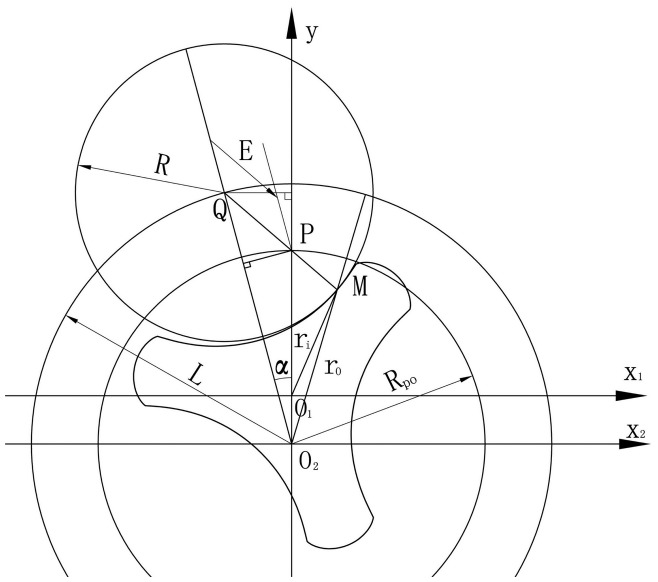
Geometric relationships of the rotors.

The instantaneous flow through the entire flowmeter *q*(*t*) is:

(8) $$q(t)=\sum_{0}^{m}\frac{\text{dv}}{\text{dt}}=\frac{\omega_{i}({r_{\text{im}}}^{2}-{r_{i0}}^{2})}{2}+\frac{\omega_{o}({r_{o0}}^{2}-{r_{\text{om}}}^{2})}{2}$$

where *m* is the number of expanding chambers. From the geometric relationships shown in Figure 7, $r_{o}^{2}$ and $r_{i}^{2}$ in Equation (8) can be expressed as:

(9) $$r_{o}^{2}=R^{2}+L^{2}-2\text{RL}(L-R_{\text{po}}\cos\alpha )/E$$

(10) $$r_{i}^{2}={\left(R_{\text{po}}-a\right)}^{2}+{\left(R-E\right)}^{2}-2(R_{\text{po}}-a)(R-E)(L\cos\alpha -R_{\text{po}})/E$$

where *E* is the length of line *PQ* (shown in Figure 7) and is defined as:

(11) $$E=\sqrt{{R_{\text{po}}}^{2}+L^{2}-2R_{\text{po}}L\cos\alpha }$$

From Equations (9) and (10), the analytical solution of the instantaneous flow through the entire flowmeter is obtained as follows:

(12) $$\begin{array}{l} q(t)=\frac{Z_{o}\omega_{o}}{2Z_{i}}\{{\left(E_{m}-R\right)}^{2}-2\left(R_{\text{po}}-a\right)\left(E_{m}-R\right)\left[R_{\text{po}}-L\cdot \cos\left(\alpha +m\lambda \right)\right]/E_{m}\\ -{\left(E_{0}-R\right)}^{2}+2\left(R_{\text{po}}-a\right)\left(E_{0}-R\right)\left(R_{\text{po}}-L\cdot \cos\alpha \right)/E_{0}\}-\frac{\omega_{o}}{2}\cdot \\ \left\{2\text{RL}\left(L-R_{\text{po}}\cdot \cos\alpha \right)/E_{0}-2\text{RL}\left[L-R_{\text{po}}\cdot \cos\left(\alpha +m\lambda \right)\right]/E_{m}\right\} \end{array}$$

Therefore, the accumulated flow *q_ac_* due to the rotations of the external rotor can be determined by integrating Equation (12) with α over the range from $\left(-\frac{\lambda }{2},+\frac{\lambda }{2}\right)$ as follows:

(13) $$q_{\text{ac}}=n\int_{-\lambda /2}^{+\lambda /2}q(t)d\alpha$$

where *n* is the number of external rotor teeth. *q_ac_* may be solved through numerical integration after the specific parameters of the flowmeter are determined. Discharge coefficient of Rotary PD meter is the volume through the flowmeter during one output pulse. So the theoretic discharge coefficient of this flowmeter can be expressed as:

(14) $$v_{t}=\frac{q_{\text{ac}}}{N_{p}}$$

where *N_P_* is the number of pulses output by the Hall sensor during one circle.

## 3. Experimental Research

**Figure 8.**
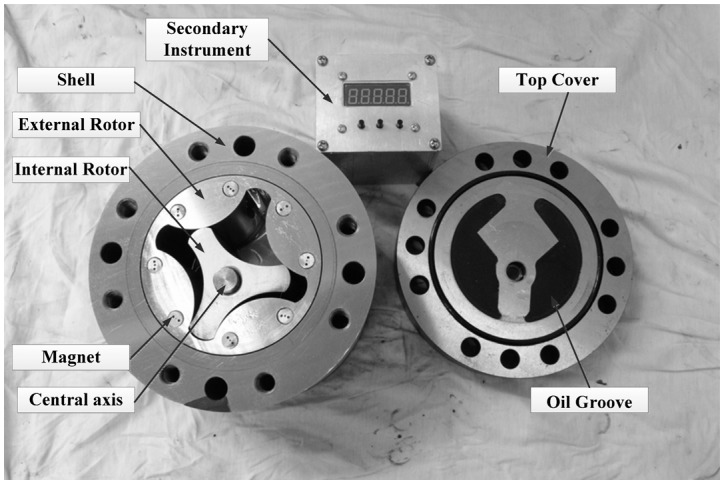
Photo of the prototype.

A cycloid rotor flowmeter was fabricated for use in the experiments described in this paper. The measuring range was designed to be 1–100 L/min. The specific parameters of the flowmeter are provided in Table 1. Figure 8 is the photo of the prototype.

### 3.1. Experimental Setup

**Figure 9.**
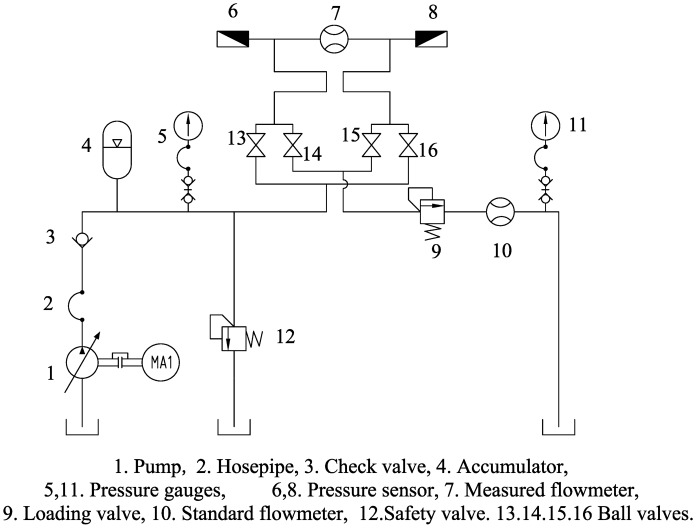
Schematic diagram of the experimental system.

An experiment was developed to test the device characteristics, as shown in Figure 9. The experimental system included a piston variable pump, a standard oval gear flowmeter, an accumulator, a safety valve, a loading valve, four ball valves, two pressure sensors with an accuracy of 0.5% and two pressure gauges with an accuracy of 0.5%. The flow rate provided by the pump can be adjusted from 0 to 120 L/min, and the system pressure can reach a maximum of 31.5 MPa. An accumulator is used to smooth the flow and pressure pulsation caused by the piston pump. Pressure gauges are used to monitor the system pressure and back pressure. The safety valve ensures that the system pressure remains under 31.5 MPa at all times. The inlet and outlet pressures of the PD meter are monitored by the pressure sensors. The loading valve is used to change the outlet pressure of the PD meter from 0 to 31.5 MPa. The four ball valves are used to change the flow direction of the fluid in the prototype. Normally calibration of PD meter uses a standard volume vessel to measure the fluid volume through the flowmeter. But in this research, the calibration system uses a high accuracy standard oval gear flowmeter with the accuracy of ±0.1% to simplify the calibration process. Since the accuracy of the standard flowmeter is high enough, its readings can be regarded as the standard value without any errors. There is no bypass between the two flowmeters, so the volume of fluid through the standard flowmeter and the cycloid rotor flowmeter is same. In this experiment, 46# antiwear hydraulic oil is used as the measured mediums, and kinematic viscosity of it is 100 cst. Fluid compressibility must be considered when the flowmeter is used to measure high pressure fluids. Fluid compressibility can be reflected by the elasticity modulus. The 46# antiwear hydraulic has different elasticity moduli when it is under different pressures as shown in Table 2 [19]. According to the elasticity modulus, the fluid compressibility can be obtained as shown in Table 3 (compared with the volume at 0 Mpa). All results given in this article have considered the compressibility of the measured media.

### 3.2. Results and Discussion

#### 3.2.1. Testing of the Sensors

**Figure 10.**
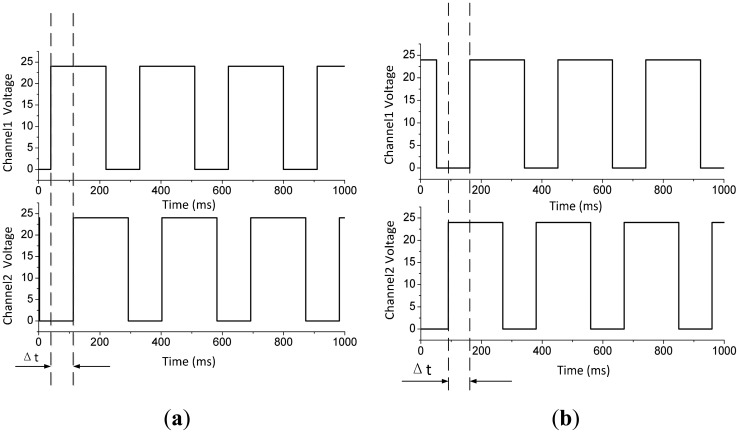
Square wave output by the Hall sensors. (**a**) Signal output by Hall sensors when oil flows forward in the flowmeter; (**b**) Signal output by Hall sensors when oil flows backward in the flowmeter.

The two square wave outputs shown in Figure 10 are produced by the two Hall sensors for a flow rate of 10.3 L/min through the standard flowmeter. When oil flows forward in the flowmeter, the output from channel 1 advances approximately π/4 radians over channel 2, as shown in Figure 10a. Conversely, the output from channel 1 lags behind channel 2 when the oil flows backwards. Thus, the sensor installed in the cycloid rotor flowmeter is working, the flow direction can be easily read from the sensor, and the flow rate can be calculated by recording the time during one pulse.

#### 3.2.2. Reading Uncertainty Analysis

**Figure 11.**
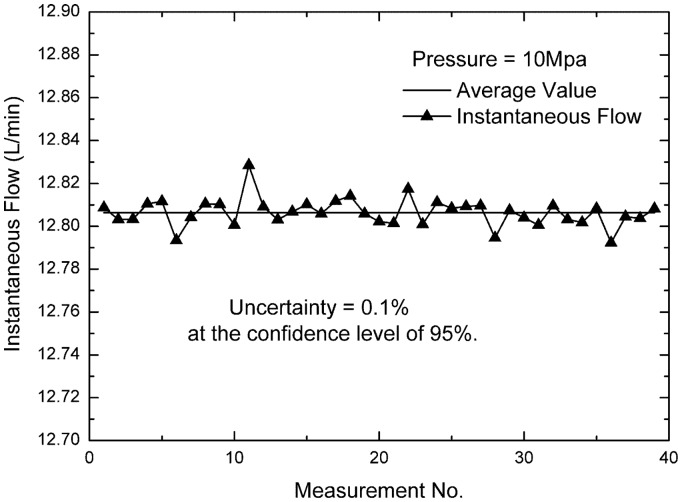
Frequent testing on the same instantaneous flow.

Reading uncertainty is an important characteristic of a measurement instrument. In order to assess the uncertainty of the cycloid rotor flowmeter, a timer is used to recording the time during 4 pulses output by the Hall sensors. The timer is triggered on the rising edge of the pulse. According to the designing parameters, the fluid volume of 4 pulses is 0.2 L, so the instantaneous flow *q_i_* can be calculated as:

(15) $$q_{i}=\frac{0.2}{\Delta t}$$

where Δ*t* is the time recording by the timer. When doing the experiment, the pressure and the instantaneous flow should be kept constant, and through frequent testing on the same instantaneous flow, the analysis assemblies are obtained. Figure 11 presents 39 measurements of the instantaneous flow at the pressure of 10 Mpa. According to a large number of testing data, the relative reading uncertainty of the cycloid rotor flowmeter is less than 0.17% at the confidence level of 95%.

#### 3.2.3. Discharge Coefficient and Relative Errors

The theoretical discharge coefficient is determined by the structure parameters of the flowmeter. The ideal discharge coefficient should be a constant and consistent with the theoretical discharge coefficient. But in fact, the real discharge coefficient is changed with the pressure and flow-rate of the fluid. Suppose the theoretical discharge coefficient is *v_t_*, and there are *N* pulses outputted by the Hall sensor, the reading value of flow is:

(16) $$Q_{m}=Nv_{t}$$

But the real value of flow observed from the standard flowmeter is:

(17) $$Q_{s}=Nv_{r}$$

where *v_r_* is the real discharge coefficient. The error is:

(18) $$\Delta Q=Q_{s}-Q_{m}=N(v_{r}-v_{t})$$

**Figure 12.**
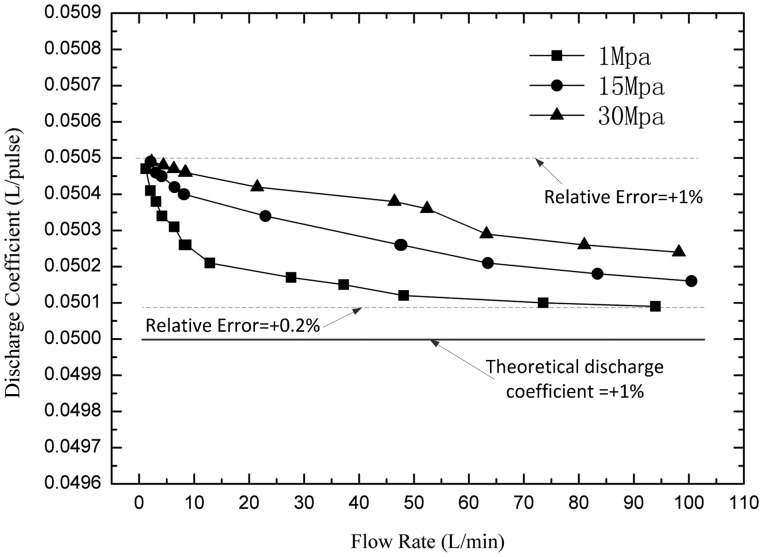
Experimental results of the discharge coefficients.

**Figure 13.**
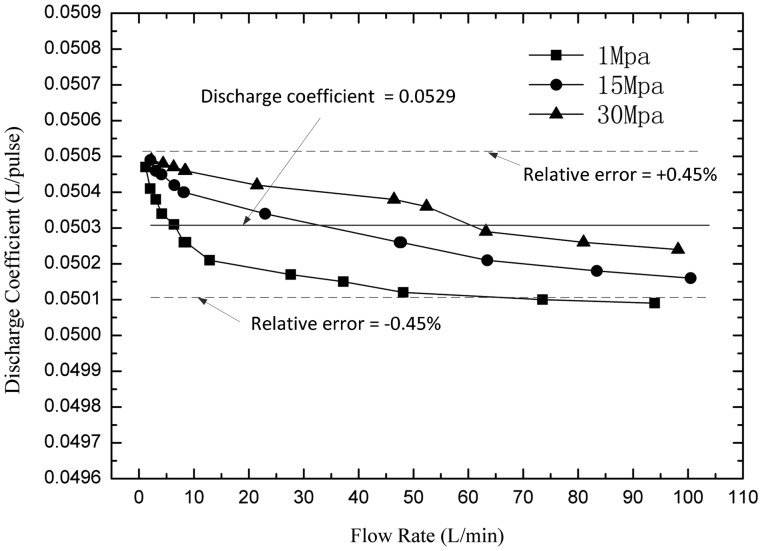
Compensated discharge coefficients according to the results.

So the change of the discharge coefficient is the major cause of error. In the experiment, the real discharge coefficients can be obtained by dividing the standard flowmeter's readings *Q_s_* by the number of pulses *N*. When doing the experiment, the instantaneous flow should be kept constant, and the total volume of the fluid through the two flowmeters should be large enough (more than 100 L) to reduce the reading errors. Figure 12 compares the real discharge coefficients with the theoretical discharge coefficients. The results suggest that all measured discharge coefficients are a little larger than the theoretical value—0.05 L/pulse, and increase with the pressure but decrease with the flow rate. The machining errors cause the integral up-down translation of all the discharge coefficients, and that can be eliminated by compensating the theoretical discharge coefficients. For example, the middle value of the biggest and smallest real discharge coefficients can be regarded as the theoretical value as shown in Figure 13, so that the relative errors can be decreased to an acceptable level.

From the results, the discharge coefficients change with pressure and flow rate. The most important reason is internal leakage. The internal leakage is a function of clearance width cubed, clearance length, viscosity, and differential pressure. The internal leakage can't be entirely eliminated, but can be limited in a certain range by decreasing the clearance and differential pressure. If a constant discharge coefficient is used to calculate the final flowrate, there will be errors due to the changes of the discharge coefficients. In this research, the relative errors are between ±0.45%.

**Figure 14.**
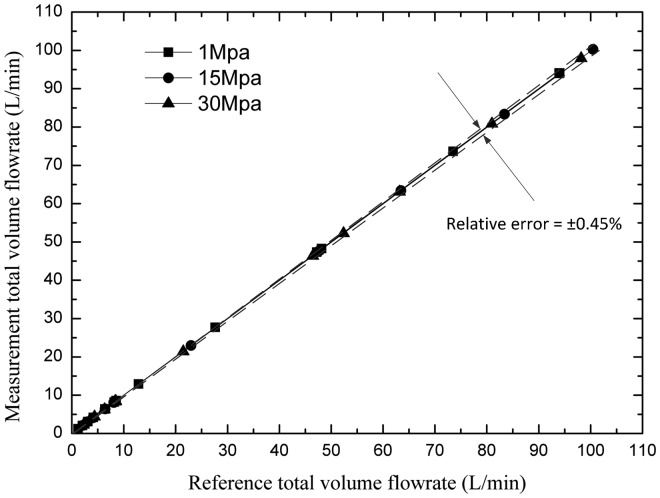
Comparison of measurements from a standard oval flowmeter and the PD meter.

Figure 14 compares a larger number of results given by the tested flowmeter with the ones given by the standard flowmeter. If all points locate at the angle bisector, it shows that there is no error. As shown, all measurement results distribute in a range with the relative errors of less than ±0.45%. From all the results and analysis, a conclusion can be obtained that the relative errors of the flowmeter under test are mainly due to the internal leakage rather than the reading uncertainty.

#### 3.2.4. Pressure Loss

**Figure 15.**
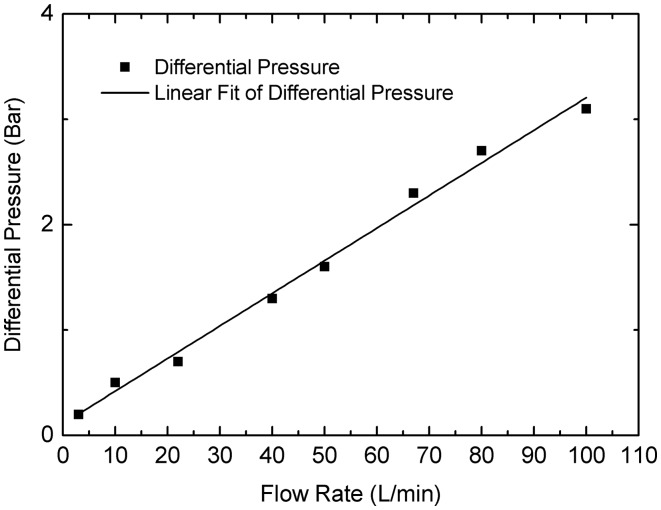
Pressure difference between the inlet and outlet.

Benefiting from the internal cycloid rotors, this flowmeter rotates smoothly without any noise. Also benefiting from the internal cycloid rotors, this flowmeter has a compact structure. It is small in size but big in displacement which is helpful to reduce the pressure loss. Pressure loss is an important characteristic of a PD meter. Larger pressure losses mean larger energy losses and internal leakage. In this experiment, two pressure sensors are connected to the inlet and outlet of the flowmeter to measure the pressure differences at different flow rates. As shown in Figure 15, the differential pressure is due to the friction between moving parts and churning losses, so it has a linear correlation with the flow rate; but nothing to do with the system pressure. Since both transverse leakage and radial clearance leakage increase with the differential pressure, the internal leakage increase obviously with the differential pressure. The differential pressure of this flowmeter is almost 3 bar when the oil flow rate is 100 L/min and the kinematic viscosity of the oil is 100 cst, which is only half as much as the differential pressure of the rotary PD meter with a pair of profile shifted gears shown in Figure 1.

## 4. Conclusions

In this work, we have discussed a rotary PD meter with a pair of internal cycloid rotors that can be widely employed for industrial automation applications. A specially designed structure and sensors are used in the flowmeter to permit high pressure resistance (up to 31.5 MPa). The cycloid rotors can mesh with each other without any assisting gears, which reduces the number of components and makes the flowmeter structure concise. The rotors with a few teeth utilize simple and easily machined shapes to reduce processing procedures and costs. Large eccentricity and few teeth make the displacement large, which is helpful to reduce the rotor speed and the pressure loss. The mathematical models of the rotor curves and the calculation method used to determine the displacement of the cycloid rotor flowmeter have been presented to help design the flowmeter.

A prototype fabricated as part of this research was able to measure bi-directional flow over ranges of 1–100 L/min with relative errors of less than ±0.5%. The rotors meshed with each other smoothly without any noise. Fluid flowing in the device causes two square waves to be output from the sensor, which directly correspond to the state of the flow rate. Furthermore, the flow direction can be determined from the phase difference between the two square waves. The experimental results demonstrate that for a flow rate of 100 L/min, the pressure loss is about 3 bar with the accuracy of ±0.5%, and the pressure loss is much lower than that of a common PD meter with profile shifted gears. In view of the limited machining ability, the internal leakage is still a little large. If the processing accuracy is further improved, the cycloid rotor flowmeter can likely achieve higher measurement accuracy and larger measurement range with less pressure loss.

## Figures and Tables

**Table 1.** Cycloid rotor flowmeter design.

Name	Value	Unit
Number of Internal Rotor Teeth	3	
Number of External Rotor Teeth	4	
Angle between the Two Hall Sensors	22.5	Degree
Eccentricity	9.3	mm
Radius of the Tooth Circle	28.6	mm
Radius of the Generating Circle	50	mm
Height of the Rotors	61.7	mm
Displacement	0.2	L/round
Discharge coefficient	0.05	L/pulse
Number of Permanent Magnets	4	Pairs
Number of Pulses	2000	Pulses/L

**Table 2.** The elasticity moduli of 46# antiwear hydraulic oil.

Pressure (MPa)	0.2	0.4	0.6	0.8	1	2
Elasticity Modulus (MPa)	580.1	757.1	876.2	957.9	1028.3	1194.0
Pressure (MPa)	3	4	5	7	9	11
Elasticity Modulus (MPa)	1252.3	1303.6	1346.7	1401.2	1426.2	1455.8
Pressure (MPa)	13	15	20	25	30	
Elasticity Modulus (MPa)	1472.3	1488.6	1537.6	1557.8	1579.4	

**Table 3.** Compressibility of the oil.

Pressure(MPa)	1 MPa	15 MPa	30 MPa
Compressibility	0.012%	1.172%	2.142%

## Nomenclature

*a*: Eccentricity of the internal and external rotors
*L*: Length of the line *O*_2_*A* in Figure 5
*R*: Radius of the tooth circle in Figure 5
φ_1_,: The base circle turning angle in Figure 5
φ_2_,: The moving circle turning angle in Figure 5
*P*: Pressure in one flowmeter chamber
*M_o_*,*M_i_*: Torque applied to the external and internal rotors by the oil
*v*: Volume of one chamber in Figure 6
*t*: Time
*r_o_*,*r_i_*: Equivalent force arm of the external and internal rotors in Figure 6
ω*_o_*,ω*_i_*: Angular velocity of the external and internal rotors
*Z_o_*,*Z_i_*: The number of external and internal teeth
*R_po_*: The radius of the external rotor pitch circle, *R_po_*=*Z_o_a*
*α*: The external rotor turning angle from the initial position in Figure 7
*λ*: The angle between two adjacent teeth of the external rotor, $\lambda =\frac{2\pi }{Z_{o}}$
